# Comparative study of effectiveness and safety between non-Descemet stripping endothelial keratoplasty and Descemet stripping endothelial keratoplasty for endothelial decompensation

**DOI:** 10.3389/fmed.2025.1499422

**Published:** 2025-05-20

**Authors:** Minghai Huang, Guina Yin, Thuthuy Hoang, Zhifeng Wu, Jian Teng, Yanqing Liang, Zhuoyuan Zhang, Dongmei Wei

**Affiliations:** Nanning Aier Eye Hospital, Nanning, China

**Keywords:** Descemet stripping endothelial keratoplasty, non-Descemet stripping endothelial keratoplasty, endothelial decompensation, endothelial keratoplasty, treatment

## Abstract

**Purpose:**

To compare the potential effectiveness and safety of non-Descemet stripping endothelial keratoplasty (nDSEK) and Descemet stripping endothelial keratoplasty (DSEK) in treating endothelial decompensation.

**Methods:**

A retrospective comparative analysis was conducted on patients with endothelial decompensation who underwent either nDSEK or DSEK procedures between August 2017 and January 2024. Participants were observed for a minimum duration of 12 months. The study documented key variables like best corrected visual acuity (BCVA), endothelial cell density (ECD), endothelial cell loss (ECL), and any issues that occurred during the follow-up period.

**Results:**

A total of 85 eyes from 85patients (nDSEK *n* = 40 eyes, DSEK *n* = 45 eyes) were ultimately included in the study for analysis based on the inclusion and exclusion criteria. The mean BCVA (logMAR) showed significant improvement from the preoperative measurement of 1.66 ± 0.26 to 0.37 ± 0.11 in nDSEK eyes and from the preoperative 1.68 ± 0.24 to 0.36 ± 0.10 in DSEK eyes, respectively, at postoperative 12 months. However, there was no statistically significant difference in the improvement of BCVA between the nDSEK and DSEK eyes (*p* = 0.605). The mean donor ECD decreased from the preoperative 2,814 ± 85 cells/mm^2^ to 1,195 ± 216 cells/mm^2^ (ECL 57.5%) in nDSEK eyes and from the preoperative 2,889 ± 125 cells/mm^2^ to 1,266 ± 285 cells/mm^2^ (ECL 56.2%) in DSEK eyes, respectively, at postoperative 12 months, with no significant difference between the nDSEK and DSEK eyes (*p* = 0.192). The occurrence of various complications (e.g., graft dislocation, acute hypertension, primary graft failure, graft rejection) was comparable between nDSEK and DSEK eyes.

**Conclusion:**

nDSEK eliminated the descemetorhexis step but yielded a comparable clinical outcome in effectiveness and safety compared to DSEK for treating endothelial decompensation.

## Introduction

Endothelial keratoplasty (EK) offers more benefits than penetrating keratoplasty (PK) in treating endothelial decompensation. It provides accelerated vision recovery and reduces graft rejection problems. Over the past two decades, EK has evolved significantly through the utilization of different techniques, such as Descemet stripping endothelial keratoplasty (DSEK), Descemet stripping automated endothelial keratoplasty (DSAEK), femtosecond laser-assisted DSEK (FS-DSEK), and Descemet membrane endothelial keratoplasty (DMEK). However, it has not been popularized in developing countries like China, which can be attributed to factors like the high cost of instruments required for procedures like DSAEK and FS-DSEK. DMEK is becoming increasingly popular and recommended as a preferred treatment, however, the technique of DMEK presents difficulties, including a high level of complexity, steep learning curve, and specific obstacles, such as the meticulous preparation of the donor Descemet membrane without any wastage. Therefore, considering the constraints of limited resources, it remains reasonable to conduct DSEK as a viable therapy for endothelial decompensation.

In Western countries, Fuchs’ endothelial dystrophy is one of the leading cause of EK, particularly among Caucasians. In contrast, post-cataract surgery endothelial decompensation is more common in China, reflecting regional and ethnic differences in disease patterns and surgical practices. The standard DSEK or DSAEK method removes the recipient Descemet membrane and endothelium complex (descemetorhexis step). However, to strip Descemet membrane and endothelium complex or perform a non-stripping EK is still a debate. A very few reports had documented a modified DSEK or DSAEK referred to as non-Descemet stripping endothelial keratoplasty (nDSEK or nDSAEK), in which removing the Descemet membrane and endothelium complex was no longer necessary, with favorable clinical results (1–3).

However, few studies have directly compared the clinical outcomes of the nDSEK and DSEK procedures, and further research is still needed. This study provides a detailed comparison of the possible effectiveness and safety of nDSEK versus DSEK in treating endothelium decompensation.

## Methods

### Study design and population

We reviewed 136 cases of nDSEK or DSEK for endothelial decompensation performed by Dr. Minghai Huang at Nanning Aier Eye Hospital from August 2017 to January 2024. The Medical Ethics Committee of the Nanning Aier Eye Hospital, China, approved this retrospective comparative study. This study was carried out following the principles of the Declaration of Helsinki, and all patients provided informed consent. All subjects underwent either nDSEK or DSEK. In certain special cases, nDSEK was performed in eyes with a history of PK and graft failure, as this technique minimizes the risk of prior PK wound dehiscence during the descemetorhexis procedure (4). Conversely, DSEK was applied to eyes affected by Fuchs’ endothelial dystrophy, where Descemet membrane stripping was recommended due to the presence of pathological guttata, which could potentially hinder visual improvement (5). The inclusion criterion was the presence of vision impairment caused by endothelial decompensation. Exclusion criteria included individuals with prominent corneal scars involving the visual axis, incomplete data, a history of penetrating keratoplasty, ocular trauma, glaucoma, optic atrophy, or macular degeneration, as well as those unable to complete a minimum follow-up period of 12 months. Information related to demographics, visual acuity, corneal endothelial cell count, and postoperative complications were collected from the medical records.

### Surgical procedure

The Donor corneas were prepared manually according to the technique described previously by Price et al. (6). The surgery was carried out using either general anesthesia or retrobulbar block anesthesia with a 50% mixture of lidocaine (2%) and bupivacaine (0.5%). A 4.0-mm superior or temporal scleral tunnel incision was created according to the different eye conditions. The central epithelium of the cornea was removed to provide a clear view of the anterior chamber, and then descemetorhexis was performed using a reverse Sinsky hook during DSEK. With cohesive viscoelasticity, descemetorhexis was performed, namely the Descemet membrane and endothelium complex were gently stripped from the central region and removed from the anterior chamber. Descemetorhexis was not used in nDSEK, in contrast to DSEK. If the eyes had not previously undergone a peripheral iridectomy, a regular surgery of peripheral iridectomy was conducted at six o’clock to avoid pupillary obstruction. As described earlier by Hong et al. (7), a suture pull-through technique was applied for donor insertion. The donor lenticule was then secured against the host cornea using a full intracameral sterile air tamponade. In eyes with severe cataracts, we conducted nDSEK or DSEK, phacoemulsification, and implantation of an intraocular lens. We used a sutureless scleral fixation technique or transscleral suture fixation to implant a foldable intraocular lens in the posterior chamber for aphakic eyes. If necessary, we conducted limited anterior vitrectomy simultaneously in cases with minimal or no capsular support. Large iris defects in the eyes were treated with DSEK or DSEK with pupilloplasty.

During follow-up visits, all the patients were examined using a slit lamp (Topcon Corporation, Tokyo, Japan) along with anterior segment optical coherence tomography (AS-OCT, Heidelberg Engineering GmbH, Germany). The study assessed the uncorrected visual acuity (UCVA), best-corrected visual acuity (BCVA), and endothelial cell density (ECD) using different equipment at different time intervals (3 months, 6 months, and 12 months postoperatively). The donor ECD was examined before surgery by EB-3000 XYZ, HAI Laboratories Inc., Lexington, MA, and after surgery by Tomey EM-4000, Tomey Co., Nagoya, Japan. For statistical analysis, decimal VA was converted into a logarithm of minimal angle of resolution (logMAR), with counting fingers being 2.0 logMAR and hand movements being 2.3 logMAR (8). Eyes with primary or secondary graft failure were excluded from the BCVA analysis, while eyes with primary or secondary graft failure and those that underwent rebubbling due to graft dislocation were excluded from the ECD analysis.

The statistical analyses were conducted using IBM SPSS Statistics, version 22 (IBM Corp., Armonk, NY, United States). The values have been shown as mean±standard deviation. A statistical analysis compared the two groups’ demographic features and baseline clinical data. This study utilized either the Student’s t-test or the Chi-square test. The alterations in BCVA and endothelial cell density (ECD) over time in the two groups were evaluated using a repeated measures analysis of variance (ANOVA) using a general linear model. Statistical significance was set at *p* < 0.05.

## Results

### Demographic characteristics and baseline clinical data of patients

A total of 136 eyes from 136 patients who underwent endothelial keratoplasty were reviewed during the study period. Based on the exclusion criteria, 51 eyes were excluded for the following reasons: corneal scars involving the visual axis (*n* = 12), incomplete data (*n* = 16), prior penetrating keratoplasty (*n* = 6), glaucoma (*n* = 12), macular degeneration (*n* = 3), and optic atrophy (*n* = 2). Ultimately, 85 eyes from 85 patients were included in the analysis, comprising 40 eyes in the nDSEK group and 45 eyes in the DSEK group. The follow-up period ranged from 12 to 36 months, with a mean of 19 ± 8 months. Patients in the nDSEK group ranged in age from 31 to 87 years old, with a mean age of 60.3 ± 15.1 years. The DSEK group’s mean age was 64.1 ± 13.6 years, ranging from 32 to 93 years. The proportion of females in the nDSEK group was 52.5%, while in the DSEK group, it was 60% (Table 1). The frequent indications included endothelial decompensation after cataract surgery, such as pseudophakic (68 eyes, 80%; nDSEK *n* = 33; DSEK *n* = 35), aphakic bullous keratopathy (5 eyes 5.9%; nDSEK *n* = 3; DSEK = 2) as well as other less common etiologies, such as Fuchs’ endothelial dystrophy (4 eyes 4.7%; DSEK *n* = 4), corneal endotheliitis (4 eyes 4.7%; nDSEK *n* = 2; DSEK *n* = 2), unknown etiology (4 eyes, 4.7%; nDSEK *n* = 2; DSEK *n* = 2) as shown in Table 1. There were no statistically significant differences found in the data related to age (*p* = 0.216), sex (*p* = 0.486), indications (*p* = 0.304), preoperative BCVA (*p* = 0.767), and ECD (*p* = 0.056) (Table 1).

**Table 1:** Demographic characteristics and baseline clinical data of patients.

	nDSEK (*n* = 40)	DSEK (*n* = 45)	*p* value
Age (years)	60.3 ± 15.1	64.1 ± 13.6	0.216
Female	21(52.5%)	27(60%)	0.486
Preoperative BCVA (LogMAR)	1.67 ± 0.26	1.69 ± 0.24	0.767
Donor ECD (cells/mm^2^)	2,826 ± 103	2,875 ± 124	0.056
Indications			0.304
Decompensation after cataract surgery	36(90%)	37(82.2%)	
Other etiologies	4(10%)	8(17.8%)	

nDSEK, non-Descemet stripping endothelial keratoplasty; DSEK, Descemet stripping endothelial keratoplasty; BCVA, best corrected visual acuity; LogMAR, logarithm of minimal angle of resolution; ECD, endothelial cell density; decompensation after cataract surgery including pseudophakic (nDSEK *n* = 33; DSEK *n* = 35) and aphakic bullous keratopathy (nDSEK *n* = 3; DSEK = 2); other etiology including Fuchs’ endothelial dystrophy (DSEK *n* = 4), corneal endotheliitis (nDSEK *n* = 2; DSEK *n* = 2), unknown etiology (nDSEK *n* = 2; DSEK *n* = 2).

### General observation

**Figure 1:**
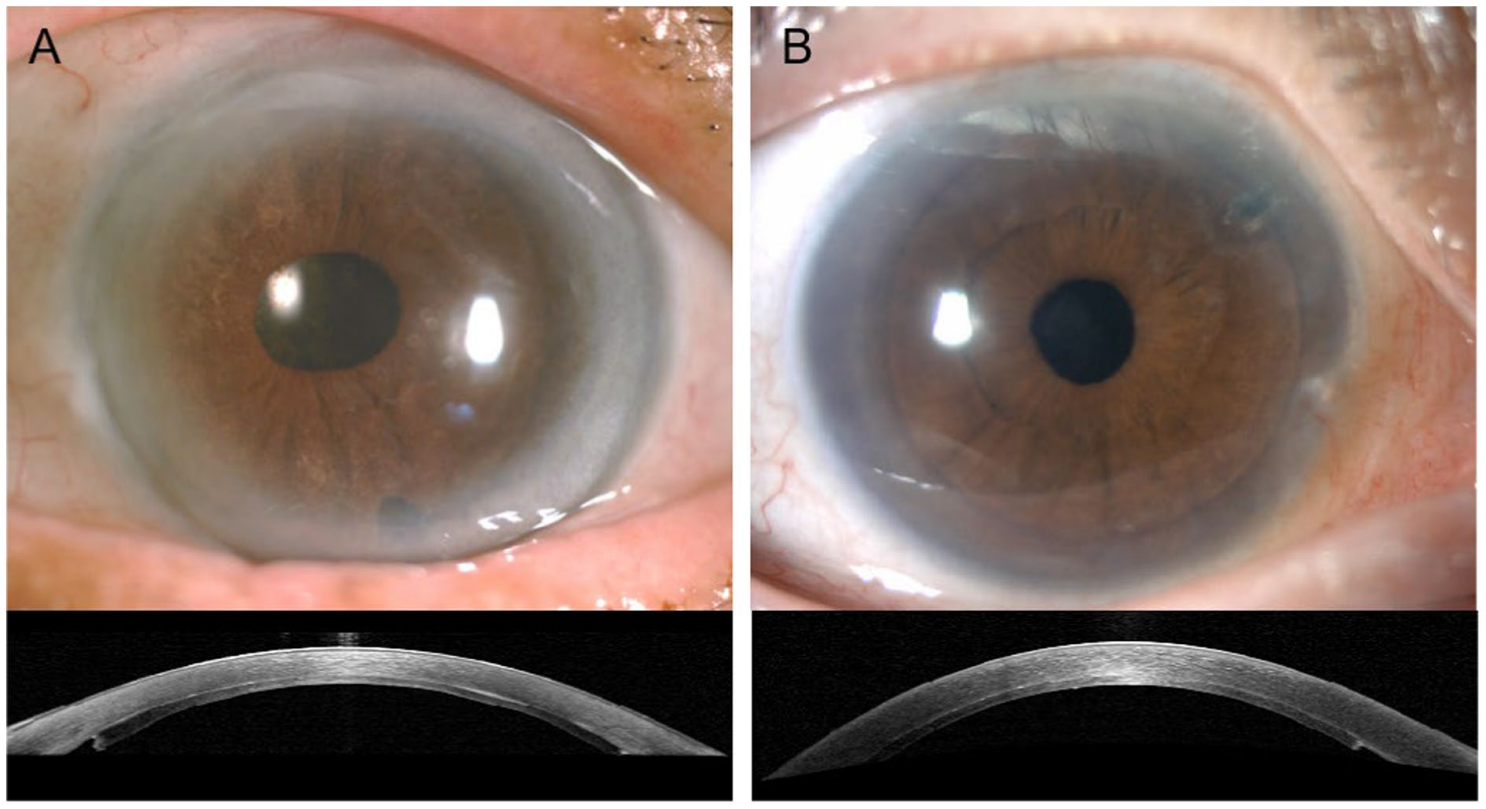
Representative slit-lamp microscope and AS-OCT photographs after completion of successful surgery. The cornea appeared transparent with no visible differences between the nDSEK **(A)** and DSEK **(B)** eyes (when examined using a slit-lamp. top), donor lenticule adhered to recipient bed well (AS-OCT, bottom); nDSEK, non-Descemet stripping endothelial keratoplasty; DSEK, Descemet stripping endothelial keratoplasty.

All patients experienced pain relief and felt comfortable with the disappeared corneal edema and bullae within 1 ~ 3 months after the successful surgery. Examined under a slit-lamp microscope, the transparent corneas of the eyes that had DSEK and nDSEK treatments showed no apparent differences. The recipient bed and donor lenticule were also firmly fixed. Representative slit-lamp microscope and AS-OCT photographs are shown in Figure 1.

### Visual outcomes

**Figure 2:**
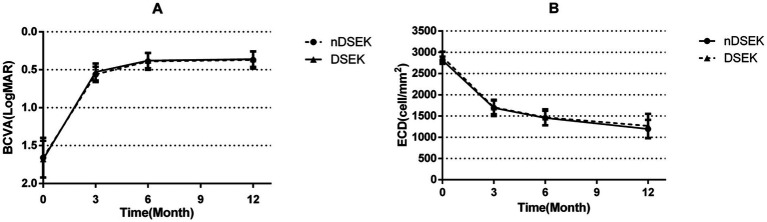
The comparison of BCVA and ECD after surgery shows variations between nDSEK and DSEK eyes at different time intervals. **(A)** BCVA was found to be improved and **(B)** ECD decreased with time, but there were no statistically significant differences observed for BCVA (*p* = 0.605) and ECD (*p* = 0.192) between nDSEK and DSEK eyes at the same time point. BCVA, best-corrected visual acuity; LogMAR, logarithm of minimal angle of resolution; ECD, endothelial cell density;nDSEK, non-Descemet stripping endothelial keratoplasty; DSEK, Descemet stripping endothelial keratoplasty.

Data analysis on BCVA excluded eyes with primary or secondary failure (nDSEK: n = 4; DSEK: n = 6) The visual results demonstrated a steady enhancement in the months after the surgical procedure, steadily advancing until the 6-month threshold for most nDSEK and DSEK eyes. The mean postoperative BCVA (logMAR) significantly improved from the preoperative 1.66 ± 0.26 to 0.56 ± 0.10 at postoperative 3 months, 0.39 ± 0.11 at postoperative 6 months, 0.37 ± 0.11 at postoperative 12 months, respectively in nDSEK eyes. In addition, the mean postoperative BCVA (logMAR) improved from the preoperative 1.68 ± 0.24 to 0.53 ± 0.11 at postoperative 3 months, 0.38 ± 0.10 at postoperative 6 months, 0.36 ± 0.10 at postoperative 12 months, respectively, in DSEK eyes. However, no statistically significant differences were observed between the nDSEK and DSEK eyes regarding statistical significance at the respective time intervals (*p* = 0.605; Table 2; Figure 2).

**Table 2:** Changes in BCVA and ECD at different time points after the surgery.

	nDSEK (n)	DSEK (n)	*p* value
Changes in BCVA			0.605
Preoperative BCVA (LogMAR)	1.66 ± 0.26 (36)	1.68 ± 0.24 (39)	
BCVA at 3 months (LogMAR)	0.56 ± 0.10 (36)	0.53 ± 0.11 (39)	
BCVA at 6 months (LogMAR)	0.39 ± 0.11 (36)	0.38 ± 0.10 (39)	
BCVA at 12 months (LogMAR)	0.37 ± 0.11 (36)	0.36 ± 0.10 (39)	
Changes in ECD			0.192
Preoperative ECD (cells/mm^2^)	2,814 ± 85 (35)	2,889 ± 125 (37)	
ECD at 3 months(cells/mm^2^)(ECL%)	1,691 ± 193(39.9%) (35)	1704 ± 160(41.0%) (37)	
ECD at 6 months(cells/mm^2^)(ECL%)	1,455 ± 169(48.3%) (35)	1,469 ± 191(49.3%) (37)	
ECD at 12 months(cells/mm^2^)(ECL%)	1,195 ± 216(57.5%) (35)	1,266 ± 285(56.2%) (37)	

nDSEK, non-Descemet stripping endothelial keratoplasty; DSEK, Descemet stripping endothelial keratoplasty; BCVA, best-corrected visual acuity; LogMAR, logarithm of minimal angle of resolution; ECD, endothelial cell density; ECL, endothelial cell loss. BCVA data excluded eyes with primary or secondary graft failure (nDSEK: *n* = 4; DSEK: *n* = 6); ECD data excluded eyes with primary or secondary graft failure and dislocation (nDSEK: *n* = 5; DSEK: *n* = 8).

### Endothelial cell density and endothelial cell loss (ECL)

Data analysis on ECD excluded eyes with primary or secondary graft failure and dislocation (nDSEK: *n* = 5; DSEK: *n* = 8) After the surgery, the mean donor ECD decreased from the preoperative 2,814 ± 85 cells/mm^2^ to 1,691 ± 193 cells/mm^2^(ECL 39.9%)at postoperative 3 months postoperative,1,455 ± 169 cells/mm^2^ (ECL 48.3%)at 6 months postoperative and 1,195 ± 216 cells/mm^2^(ECL 57.5%) at 12 months postoperative, respectively, in nDSEK eyes. Similarly, in DSEK eyes, the mean donor ECD decreased from the preoperative 2,889 ± 125 cells/mm^2^ to 1704 ± 160 cells/mm^2^ (ECL 41%) at 3 months postoperative, 1,469 ± 191 (ECL 49.3%) cells/mm^2^ at 6 months postoperative and 1,266 ± 285 cells/mm^2^ (ECL 56.2%) at 12 months postoperative, respectively. However, no statistically significant differences were found between the nDSEK and DSEK eyes at the same time points (*p* = 0.192; Table 2; Figure 2).

### Complications

Three eyes (7.5%) experienced graft dislocation following nDSEK, while three eye (6.7%) experienced graft dislocation after DSEK, specifically on postoperative days 1 to 2. Nevertheless, successful rebubbling was performed in all of these cases. The graft dislocation rates showed no significant differences between nDSEK and DSEK eyes (*p* = 0.881; Table 3). Acute high intraocular pressure occurred in two eye (5%) after nDSEK and four eyes (8.9%) after DSEK, respectively even routinely had Peripheral iridectomy. The nDSEK group (*n* = 2) exhibited a mean IOP elevation of 48.9 mmHg (range: 42.4–55.3), while the DSEK group (*n* = 4) showed a mean elevation of 43.2 mmHg (range: 36.6–50.6). The problem was resolved by eliminating extra air that caused an obstruction in the pupil or by performing angle reformation due to secondary angle closure resulting from air migration behind the iris between the first and second day after the procedure. No statistically significant differences were found in the acute high intraocular pressure rate between the nDSEK and DSEK eyes (*p* = 0.485; Table 3). None of the patients with temporary acute high intraocular pressure elevation developed glaucoma during the study period. Three eyes (7.5%) experienced primary graft failure following nDSEK, while four eyes (8.9%) experienced it after DSEK. Except for one DSEK eye that needed penetrating keratoplasty a year after the first procedure, all these eyes received re-grafting employing fresh endothelium donor tissue within 6 months. However, no statistically significant variations existed between the nDSEK and DSEK eyes’ primary graft failure rates (*p* = 0.816; Table 3). Graft rejection was observed in 5% of eyes following nDSEK, while only 4.4% of eyes experienced rejection after DSEK. However, the study found no significant differences in endothelial graft rejection between the nDSEK and DSEK eyes (*p* = 0.904; Table 3). All these rejection cases were managed with frequent topical prednisolone acetate eye drops and subconjunctival dexamethasone injections. One nDSEK eye responded favorably, but the other patients eventually had secondary graft failure.

**Table 3:** Complications observed after the surgery.

	nDSEK (*n* = 40)	DSEK (*n* = 45)	*p* value
Graft dislocation	3(7.5%)	3(6.7%)	0.881
Acute high introcular pressure	2(5%)	4(8.9%)	0.485
Primary graft failure	3(7.5%)	4(8.9%)	0.816
Graft rejection	2(5%)	2(4.5%)	0.904

nDSEK, non-Descemet stripping endothelial keratoplasty; DSEK, Descemet stripping endothelial keratoplasty.

## Discussion

The results of our study showed that both the nDSEK and DSEK groups experienced substantial and similar improvements in BCVA. The enhancement of BCVA seen in this study was similar to the findings reported in prior studies. Zhang’s study (2) found that following nDSEK, the average BCVA (logMAR) increased from 1.70 before the surgery to 0.54 at 3 months, 0.46 at 6 months, and 0.37 at 1 year post-surgery. Moreover, in a short-term comparative study by Mohamed et al. in pseudophakic corneal edema (9), the mean BCVA (logMAR) at 6 months postoperatively in the DSEK and nDSEK eyes were found to be 0.18 and 0.44, respectively, with no significant difference observed between them. In a recent study carried out by Omoto et al. (10), the long-term outcomes of DSAEK were compared to those of nDSAEK. The study found that the mean preoperative BCVA (logMAR) of nDSAEK and DSAEK eyes were 1.08 and 1.11, respectively. However, these values significantly improved to 0.238 and 0.190, 0.126 and 0.157, and 0.097 and 0.070 at 1, 3, and 5 years, respectively. No significant statistical differences were observed in the improvement of BCVA between nDSAEK and DSAEK procedures. This suggests that removing the recipient’s Descemet membrane may not be necessary and has minimal impact on long-term outcomes.

We concluded that multiple factors contributed to the lower level of visual improvement in our study compared to the previous studies. For example, the mean preoperative BCVA was very poor. One possible explanation could be the result from prolonged corneal edema caused by the extended waiting period for the surgery. While Fuchs’ endothelial dystrophy is one of the leading indications for keratoplasty in Western countries (11, 12), post-cataract surgery endothelial decompensation, including pseudophakic and aphakic bullous keratopathy, is more common in China. The clinical outcomes, such as postoperative vision improvement, were similarly impacted by these problems. In the context of Fuchs’ endothelial dystrophy, nDSEK is not recommended due to the pathological guttata potentially affecting the visual improvement (5). However, it is worth noting that Fuchs’ endothelial dystrophy is less common in China compared to Europe and the US. Other cases, such as those with significant Descemet’s membrane abnormalities and retro corneal fibrous membrane, are unsuitable for nDSEK and should perform the descemetorhexis procedure. The nDSEK offers a smoother learning process for inexperienced surgeons, as it eliminates the need for descemetorhexis. This helps prevent unintended complications, such as unintentional damage to the posterior corneal stroma and accidental displacement of the recipient’s Descemet membrane fragments into the posterior segment, particularly in cases where there is no lens-iris diaphragm. In addition, nDSEK may also benefit cases in two specific scenarios: first, for those with a history of prior PK, as it eliminates the need for descemetorhexis and thereby reduces the likelihood of prior PK wound dehiscence during surgery (4); second, for cases with congenital hereditary endothelial dystrophy, where Descemet membrane removal is technically challenging due to difficult manipulation and poor surgical visibility (13).

Our study showed a significant decrease in donor endothelial cell density (ECD) in both nDSEK and DSEK eyes at various postoperative time points. However, there were no statistically significant differences in ECD between nDSEK and DSEK eyes simultaneously. Interestingly, the ECL in the present study’s nDSEK and DSEK eyes was higher than those reported in the previous studies. For instance, in Price’s study on DSEK, the ECL was 34% at 6 months, 36% at 12 months, and 41% at 24 months (14). Moreover, in Mohamed’s study, the ECL in the DSEK and nDSEK eyes was 28.1 ± 17.1 and 23.6% ± 8.3%, respectively (9). We postulate that several factors may have contributed to the higher endothelial cell loss in our current study after nDSEK and DSEK treatments. Firstly, endothelial keratoplasty may be more difficult in these complex cases if there is a history of cataract, eyes with severe corneal edema; or abnormal anterior segments, such as anterior synechia, aphakia, and iris defect. Moreover, many parameters about the surgical procedure itself, such as the size of the incision, the preparation of donor tissue, the use of graft delivery systems, the need for rebubbling in cases of donor tissue dislocation, and the surgeon’s learning curve in performing EK, have also been found to be associated with postoperative corneal endothelium loss (14–17).

Graft dislocation is a frequently observed complication in endothelial keratoplasty, with a dislocation rate ranging from 0 to 80% and an average rate of 14.5% (18). Several variables can affect the surgery, including the surgeon’s experience, viscoelastic in the graft interface, geometric discrepancies between the donor and recipient curvatures that cause some of the donor to arc away from the recipient, and leftover strands of either stroma or Descemet membrane that prevent the donor from being tightly apposed against the recipient (17, 19). Although the rate of graft dislocation in the nDSEK group (7.5%) was higher than that in the DSEK group (6.7%) in our study, the disparity between the two groups failed to achieve statistical significance, which suggest that retaining the recipient’s Descemet membrane has little effect on the donor lenticule’s adhesion to the recipient bed. However, this aspect may influence the rate of graft dislocation.

Acute high intraocular pressure was observed in 2 eye (5%) after nDSEK and 4 eyes (8.9%) after DSEK, despite the routine peripheral iridectomy performed. However, there were no significant statistical differences between the occurrence of this complication in nDSEK and DSEK eyes. The acute high intraocular pressure rate was relatively lower, compared to the 10.5% reported in the study conducted by Daubert et al. (17) but higher than the 2.8% reported by the study of Basak et al. (20). The increased incidence of immediate high intraocular pressure in our current study may be linked to applying a complete intracameral air tamponade to achieve secure attachment of the donor after the surgery without subsequent release of the air. During the follow-up period, while we observed no progression to glaucoma or obvious optic nerve and retinal damage in patients who experienced temporary acute intraocular pressure elevation, it is well known that elevated intraocular pressure produces progressive changes in the optic nerve and retina, and study have demonstrated that even transient intraocular pressure elevation can lead to damage in the optic nerve and retina (21). Therefore, releasing an appropriate amount of air at the end of surgery may help prevent postoperative acute high intraocular pressure. Close monitoring of symptoms and intraocular pressure after surgery, combined with the prompt identification and management of elevated intraocular pressure, is crucial to preventing potential irreversible damage to the optic nerve and retina.

The literature shows a broad range of primary graft failure rates, ranging from 0 to 29%, with an average rate of 5% (18). In the present study, no statistically significant differences were observed in primary graft failure between nDSEK and DSEK eyes, as three eyes (7.5%) with primary graft failure were observed in nDSEK eyes. In comparison, four eyes (8.9%) experienced primary graft failure in DSEK eyes. The primary graft failure rate in our study exceeded the percentages reported by Price, which were 5 and 6% (6, 9). Complications during anterior segment surgery can be attributed to various factors, such as the surgeon’s proficiency in performing EK, surgical cases with complex aberrant anterior segments, subpar quality of donor tissue, and inadequate donor tissue preparation (6, 22). In our study, the higher rate of primary graft failure could be attributed to the surgical procedure’s intricacy and the challenging EK learning process for surgeons.

We found graft rejection occurred in two eyes (5%) after nDSEK compared to two eyes (4.4%) after DSEK. However, no significant statistical difference in graft rejection rates was observed. The rate of graft rejection after nDSEK was found to be slightly higher than the reported rates of 4.3% by Chaurasia (4) and 3.1% by Zhang (2) after nDSEK. The factors that increase the risk of corneal rejection include host bed vascularity due to longer corneal edema in endothelial decompensation eye, clinical history of glaucoma, previous surgeries such as glaucoma surgery or anterior segment surgery, anterior iris synechiae, vitreous adhesion, re-grafts, multiple surgeries performed simultaneously (23). Furthermore, the variation in postoperative steroid administration and the length of follow-up presented difficulties in directly comparing rejection rates among various trials.

Several limitations should be acknowledged in the current study. First, its retrospective nature and relatively small sample size may limit the strength of our conclusions. This retrospective study did not perform an *a priori* sample size calculation due to inherent limitations of pre-existing data. However, a post-hoc power analysis using G*Power (effect size *f* = 0.25; *α* = 0.05; *N* = 85; 2 groups; 4 repeated measures; correlation = 0.5) demonstrated a statistical power (1-*β*) of 0.82, marginally exceeding the conventional 0.80 adequacy threshold. While sufficient for detecting moderate effects, the study may lack sensitivity to identify smaller yet clinically meaningful differences. Second, the short duration of follow-up may not fully capture long-term outcomes. Third, the heterogeneity in transplant indications and potential confounding factors, such as postoperative rebubbling, may have influenced the results. Additionally, these variables could introduce confounding bias that affects the interpretation of our findings. To address these limitations and validate our results, it is essential to conduct well-designed multi-center randomized controlled trials with a substantial number of participants and extended follow-up periods. This comprehensive approach would not only expand the participant pool but also enable the verification of findings across diverse clinical settings. Such broader validation is crucial for enhancing the reliability and generalizability of the results, thereby addressing the inherent limitations of our current retrospective study.

In conclusion, nDSEK eliminated the descemetorhexis step but yielded a clinical outcome in effectiveness and safety comparable to DSEK for treating endothelial. Decompensation. Thus, excepting patients with substantial anomalies in the Descemet membrane associated with Fuchs’ endothelial dystrophy and retro corneal fibrous membrane, we view nDSEK as a useful choice for treating endothelial decompensation.

## Data Availability

The raw data supporting the conclusions of this article will be made available by the authors, without undue reservation.
